# Supplementary material for: The Online Misinformation Susceptibility Scale: Development and Initial Validation
Source: Healthcare (Basel). 2025 Sep 8;13(17):2252. doi: 10.3390/healthcare13172252 (PMC12428072; doi:10.3390/healthcare13172252)
Supplement: Supplementary file 1 [file healthcare-13-02252-s001.zip › healthcare-3773409-Supplementary Table S8.pdf]

Supplementary Table S8. Cohen's kappa for the one-factor model with nine items for the Online Misinformation Susceptibility Scale (n=40).

**Please think about what you do when you see a post or story that interests you on social media or websites.** **How often do you …**	**Cohen's kappa**	**P-value**
1. check the website domain and URL?	0.870	<0.001
2. check the publication date of the post?	0.756	<0.001
3. check if the post includes reliable links and references such as scientific articles?	0.820	<0.001
4. check the post for grammatical, spelling, or expression errors?	0.839	<0.001
5. check if the post includes the author's name?	0.777	<0.001
6. seek more information about the author of the post?	0.767	<0.001
7. check if the post originates from a reliable source, such as authoritative news sites?	0.899	<0.001
8. check if the post is reliable by searching other reliable sources on the web?	0.732	<0.001
9. check the website design?	0.968	<0.001
