# Supplementary material for: The Online Misinformation Susceptibility Scale: Development and Initial Validation
Source: Healthcare (Basel). 2025 Sep 8;13(17):2252. doi: 10.3390/healthcare13172252 (PMC12428072; doi:10.3390/healthcare13172252)
Supplement: Supplementary file 1 [file healthcare-13-02252-s001.zip › healthcare-3773409-Supplementary Table S1.pdf]

Supplementary Table S1. The 26 items that were produced after the initial development phase of the Online Misinformation Susceptibility Scale.

Please think about what you do when you see a post or story that interests you on social media or websites.

**How often do you ...**
1. read the post in full?
2. share the post without read it in full?
3. make a comment for the post without read it in full?
4. check the website domain and URL?
5. check the publication date of the post?
6. check if the post has been updated?
7. check if the post includes reliable links and references such as scientific articles?
8. check the post for grammatical, spelling, or expression errors?
9. check if the post includes the author's name?
10. seek more information about the author of the post?
11. check if it is possible to contact the author of the post (e.g. if his/her email address is available)?
**12. search for other posts by the same author?**
13. check if the post solely expresses the opinion or experiences of the author?
14. read the comments that the post received?
15. check if the post originates from a reliable source, such as authoritative news sites?
16. check if the post is reliable by searching other reliable sources on the web?
**17. check if the post has been posted on official sources?**
18. check the website design?
**19. check what reputable sources write about the website domain?**
**20. seek more information about the owner of the website?**
21. discuss the post with someone you consider to be an expert?
**22. check if the post promotes an underlying motive?**
**23. check if the post is sponsored?**
24. use the Google image search to search if the post is true?
**25. use fact-checking websites to check whether the post is true?**
26. check if the photos and videos in the post are real?

Seven items (highlighted in bold) were removed after examination of content validity of the scale through experts' assessments (see text).
