# Supplementary material for: The Online Misinformation Susceptibility Scale: Development and Initial Validation
Source: Healthcare (Basel). 2025 Sep 8;13(17):2252. doi: 10.3390/healthcare13172252 (PMC12428072; doi:10.3390/healthcare13172252)
Supplement: Supplementary file 1 [file healthcare-13-02252-s001.zip › healthcare-3773409-Supplementary Table S2.pdf]

Supplementary Table S2. The 19 items that were remained after the assessment of the content and face validity of the Online Misinformation Susceptibility Scale.

Please think about what you do when you see a post or story that interests you on social media or websites.

**How often do you ...**
1. read the post in full?
2. share the post without read it in full?
3. make a comment for the post without read it in full?
4. check the website domain and URL?
5. check the publication date of the post?
6. check if the post has been updated?
7. check if the post includes reliable links and references such as scientific articles?
8. check the post for grammatical, spelling, or expression errors?
9. check if the post includes the author's name?
10. seek more information about the author of the post?
11. check if it is possible to contact the author of the post (e.g. if his/her email address is available)?
12. check if the post solely expresses the opinion or experiences of the author?
13. read the comments that the post received?
14. check if the post originates from a reliable source, such as authoritative news sites?
15. check if the post is reliable by searching other reliable sources on the web?
16. check the website design?
17. discuss the post with someone you consider to be an expert?

18. use the Google image search to search if the post is true?

19. check if the photos and videos in the post are real?
