# Supplementary material for: The Online Misinformation Susceptibility Scale: Development and Initial Validation
Source: Healthcare (Basel). 2025 Sep 8;13(17):2252. doi: 10.3390/healthcare13172252 (PMC12428072; doi:10.3390/healthcare13172252)
Supplement: Supplementary file 1 [file healthcare-13-02252-s001.zip › healthcare-3773409-Supplementary Table S3.pdf]

Supplementary Table S3. Inter-item correlations between the 19 items that were produced after the initial development phase of the Online Misinformation Susceptibility Scale (n=522).

**Item**	**2**	**3**	**4**	**5**	**6**	**7**	**8**	**9**	**10**	**11**	**12**	**13**	**14**	**15**	**16**	**17**	**18**	**19**
**1**	0.02	0.07	0.34	0.43	0.03	0.32	0.30	0.32	0.23	0.08	0.09	0.34	0.38	0.36	0.23	0.31	0.06	0.08
**2**		0.33	0.06	0.08	0.94	0.14	0.10	0.10	0.02	0.35	0.34	0.03	0.08	0.04	0.02	-0.04	0.97	0.32
**3**			0.04	0.05	0.36	0.09	0.05	0.06	-0.02	0.97	0.90	-0.07	0.09	-0.02	-0.01	0.01	0.32	0.92
**4**				0.62	0.08	0.68	0.49	0.53	0.42	0.04	0.03	0.32	0.63	0.58	0.48	0.40	0.07	0.03
**5**					0.09	0.64	0.57	0.57	0.42	0.06	0.05	0.35	0.60	0.53	0.38	0.34	0.09	0.06
**6**						0.15	0.10	0.10	0.02	0.39	0.37	0.02	0.09	0.06	0.03	-0.03	0.90	0.35
**7**							0.57	0.66	0.55	0.10	0.09	0.33	0.70	0.64	0.52	0.45	0.15	0.10
**8**								0.63	0.44	0.05	0.04	0.39	0.56	0.48	0.43	0.33	0.13	0.05
**9**									0.70	0.06	0.07	0.35	0.67	0.57	0.50	0.38	0.13	0.07
**10**										-0.03	-0.03	0.32	0.54	0.54	0.51	0.46	0.04	-0.02
**11**											0.93	-0.08	0.10	-0.02	-0.02	0.01	0.35	0.95
**12**												-0.07	0.09	-0.02	-0.01	0.002	0.34	0.97
**13**													0.46	0.42	0.31	0.30	0.05	-0.06
**14**														0.76	0.60	0.44	0.10	0.09
**15**															0.58	0.49	0.07	-0.02
**16**																0.52	0.04	-0.02
**17**																	-0.02	0.02
**18**																		0.32
