# Supplementary material for: The Online Misinformation Susceptibility Scale: Development and Initial Validation
Source: Healthcare (Basel). 2025 Sep 8;13(17):2252. doi: 10.3390/healthcare13172252 (PMC12428072; doi:10.3390/healthcare13172252)
Supplement: Supplementary file 1 [file healthcare-13-02252-s001.zip › healthcare-3773409-Supplementary Table S4.pdf]

Supplementary Table S4. The 12 items that were remained after the item analysis for the Online Misinformation Susceptibility Scale.

Please think about what you do when you see a post or story that interests you on social media or websites.

**How often do you ...**
1. read the post in full?
2. check the website domain and URL?
3. check the publication date of the post?
4. check if the post includes reliable links and references such as scientific articles?
5. check the post for grammatical, spelling, or expression errors?
6. check if the post includes the author's name?
7. seek more information about the author of the post?
8. read the comments that the post received?
9. check if the post originates from a reliable source, such as authoritative news sites?
10. check if the post is reliable by searching other reliable sources on the web?
11. check the website design?
12. discuss the post with someone you consider to be an expert?
