# Supplementary material for: The Online Misinformation Susceptibility Scale: Development and Initial Validation
Source: Healthcare (Basel). 2025 Sep 8;13(17):2252. doi: 10.3390/healthcare13172252 (PMC12428072; doi:10.3390/healthcare13172252)
Supplement: Supplementary file 1 [file healthcare-13-02252-s001.zip › healthcare-3773409-Supplementary Table S6.pdf]

Supplementary Table S6. The final version of the Online Misinformation Susceptibility Scale.

Please think about what you do when you see a post or story that interests you on social media or websites.

**How often do you ...**	**Never**	**Rarely**	**Sometimes**	**Very often**	**Always**
1. check the website domain and URL?	5	4	3	2	1
2. check the publication date of the post?	5	4	3	2	1
3. check if the post includes reliable links and references such as scientific articles?	5	4	3	2	1
4. check the post for grammatical, spelling, or expression errors?	5	4	3	2	1
5. check if the post includes the author's name?	5	4	3	2	1
6. seek more information about the author of the post?	5	4	3	2	1
7. check if the post originates from a reliable source, such as authoritative news sites?	5	4	3	2	1
8. check if the post is reliable by searching other reliable sources on the web?	5	4	3	2	1
9. check the website design?	5	4	3	2	1

Please, add answers on all items. Total score ranges from 9 to 45. Higher scores indicate higher misinformation susceptibility.
