# Supplementary material for: The Online Misinformation Susceptibility Scale: Development and Initial Validation
Source: Healthcare (Basel). 2025 Sep 8;13(17):2252. doi: 10.3390/healthcare13172252 (PMC12428072; doi:10.3390/healthcare13172252)
Supplement: Supplementary file 1 [file healthcare-13-02252-s001.zip › healthcare-3773409-Supplementary Table S7.pdf]

Supplementary Table S7. Corrected item-total correlations and Cronbach's alpha (when a single item was deleted) for the one-factor model with nine items for the Online Misinformation Susceptibility Scale (n=522).

Please think about what you do when you see a post or story that interests you on social media or websites. How often do you ...	Corrected item-total correlations	Cronbach's alpha (when a single item was deleted)
1. check the website domain and URL?	0.704	0.912
2. check the publication date of the post?	0.687	0.913
3. check if the post includes reliable links and references such as scientific articles?	0.800	0.905
4. check the post for grammatical, spelling, or expression errors?	0.660	0.915
5. check if the post includes the author's name?	0.769	0.907
6. seek more information about the author of the post?	0.649	0.915
7. check if the post originates from a reliable source, such as authoritative news sites?	0.815	0.905
8. check if the post is reliable by searching other reliable sources on the web?	0.743	0.909
9. check the website design?	0.625	0.917
